# Tackling Biological Risk in the Workplace: Updates and Prospects Regarding Vaccinations for Subjects at Risk of Occupational Exposure in Italy

**DOI:** 10.3390/vaccines7040141

**Published:** 2019-10-08

**Authors:** Paolo Durando, Guglielmo Dini, Emanuela Massa, Giuseppe La Torre

**Affiliations:** 1 Section of Occupational Medicine, Department of Health Sciences, University of Genoa, Via A. Pastore 1, 16132 Genoa, Italy; guglielmo.dini@unige.it (G.D.); emanuela.massa@edu.unige.it (E.M.); 2 Occupational Medicine Unit, IRCCS Ospedale Policlinico San Martino, Largo R. Benzi, 10 (Building 3), 16132 Genoa, Italy; 3 Department of Public Health and Infectious Diseases, Sapienza University of Rome, Piazzale Aldo Moro 5, 00185 Rome, Italy; giuseppe.latorre@uniroma1.it; 4 Occupational Medicine Unit, Policlinico Umberto I Hospital, Viale del Policlinico 155, 00185 Rome, Italy

**Keywords:** preventive and protective measures, occupational biological risk, vaccinations, occupational medicine, workplace

## Abstract

Occupational activities may expose workers to a variety of risks. Exposure to biological agents constitutes a traditional risk in numerous occupational settings. Legislative Decree (D.Lgs.) 81/2008 constitutes the main Italian legislative basis for the management and the prevention of biological risk in occupational settings and lists the available vaccinations against each single biological agent. The 2017–2019 National Vaccination Prevention Plan (PNPV) identifies some categories of workers for whom specific vaccinations are indicated. In this context, the occupational physician identifies work processes that are at risk—identifying susceptible workers and providing information on health monitoring—and is responsible for ensuring that vaccinations are carried out. Adequate and thorough evaluation of risk are indispensable to appropriate consultation by the occupational physician in order to enable the employer to provide efficacious vaccinations. Close collaboration among the services of occupational medicine, vaccination clinics, and healthcare management together with the implementation of vaccination programs that are agreed upon at the institutional level provides an opportunity to reduce the number of workers who are susceptible to vaccine-preventable diseases, thereby yielding benefits in terms of biological risk management in the workplace and contributing to increasing vaccination coverage rates, which in many cases are currently unsatisfactory.

## 1. Introduction

Occupational activities may expose workers to a variety of risks, which can be identified on the basis of the type of working environment and the specific tasks carried out, representing health and safety threats. Exposure to biological agents constitutes a traditional risk in numerous occupational settings (e.g., health care, agriculture, the modern biotechnology industry) and is regulated by law [1]. According to the data reported by Hamalainen et al., each year, about 2.78 million work-related deaths are estimated to occur worldwide. Of these, about 9% are attributable to transmissible diseases, which account for about 30% of work-related deaths in low- and middle-income countries and for less than 5% in high-income countries [2].

The term “biological risk” refers to the probability of contracting a disease (mainly infectious, but possibly also allergic or toxic) following exposure to biological agents, microorganisms, or cell cultures. Sections X and X-bis of Legislative Decree 81/2008 (D.Lgs. 81/2008) constitute the main legislative basis for the management and the prevention of biological risk in occupational settings [3].

According to the definition provided by Art. 267 of D.Lgs. 81/2008 and subsequent amendments, a biological agent is defined as “any microorganism, even if genetically modified, cell culture or human endoparasite capable of causing infection, allergy or intoxication” [3].

Moreover, this legislation also requires that all risks for the health of workers be properly evaluated, which is a prerequisite to identifying and implementing appropriate measures of prevention and protection. With regard to biological risk, such measures include the provision of efficacious vaccines by the employer on the advice of the occupational physician for all workers who are not immune to the biological agent present in the production process as well as for individual health reasons [3]. However, assessing biological risk and its effects accurately can be challenging, mainly because no precise limit to occupational exposure has been defined [1].

The magnitude of the risk due to exposure to biological agents is commensurate with the gravity of the disease caused by the specific agent (harmful potential) and with certain intrinsic characteristics of the biological agent (infectivity, pathogenicity, transmissibility, neutralizability), which determine its ability to penetrate the human organism and to cause various degrees of damage (Table 1).

The ANNEXE XLVI of D.Lgs. 81/2008 reports the list of biological agents that are classified in groups 2, 3, and 4. This list considers the effects of these biological agents on healthy workers; it does not take into account other factors, such as underlying disorders, the use of medicines, immune deficiency, pregnancy, or breastfeeding, which might modify the individual’s sensitivity to the biological agents.

Biological agents that represent a health and safety threat may be present in numerous occupational settings, and the modalities of transmission of occupational infections may differ according to the work tasks carried out, the features of the workplace, and the microorganisms involved. Thus, during the assessment of biological risk, one may observe: (i) a generic biological risk that is potentially present in all workplaces; and (ii) a specific biological risk pertaining to the task performed.

Specific biological risks arise in activities in which biological agents are intentionally used as an integral part of the process of production or handling. These activities are exemplified in ANNEXE XLIV of D.Lgs. 81/2008 (e.g., the food industry; agriculture; zootechnics; butchery; fish farming; veterinary services; the processing of animal-derived products such as leather, skins, wool, etc., healthcare services such as hospitals, outpatient clinics, dental surgeries, and patient-care services; mortuary and cemetery services; waste collection, treatment, and disposal; industrial facilities for the sterilization, disinfection, and cleansing of potentially contaminated materials; waste-water purification and the maintenance of sewers) [3].

In addition, D.Lgs. 81/2008 constitutes the principal legislative reference for the provision of efficacious vaccines by employers; ANNEXE XLVI contains a list, updated to 2008, of the vaccinations available for individual biological agents [3].

With regard to the management and the prevention of biological risk in the healthcare setting, the “Guidelines for the health surveillance of healthcare workers exposed to biological risk” issued by the Italian Society of Occupational Medicine and Industrial Hygiene [SIMLII; since 2017, Italian Society of Occupational Medicine (SIML)] identified some areas of paramount importance as long ago as 2005. These are still of extreme relevance to the occupational physician today and are also largely applicable to other occupational settings (e.g., risk assessment, accidents involving biological risk, role of the occupational physician, health surveillance, mandatory and recommended vaccinations, equivalent persons, and the evaluation of fitness, with reference both to the protection of the worker’s health and to the prevention of risks for patients resulting from a worker affected by a transmissible disease or infection). [4]

With regard to the specific contribution of vaccinations to the prevention of biological risk in the workplace, the 2017–2019 National Vaccination Prevention Plan (PNPV) reports indications regarding the need to implement adequate vaccination programs. These are aimed at substantially reducing the risk of acquiring dangerous occupational infections and of transmitting pathogens to other workers or persons with whom the worker may come into contact (e.g., patients, relatives). A primary objective of the plan is to harmonize vaccination strategies nationwide in order to ensure that the entire population can benefit from vaccination, which is seen as a means of both individual protection and collective prevention. In addition, the plan identifies some categories of workers for whom specific vaccinations are indicated [5].

## 2. Tasks and Role of the Occupational Physician in Vaccine Prevention

Within the framework of health surveillance, the occupational physician, in accordance with Art. 41 of D.Lgs. 81/2008, provides health services aimed at safeguarding the health and the psycho-physical well-being of workers and equated subjects. This is done through the implementation of protocols drawn up on the basis of specific risks identified in the workplace; these protocols are periodically updated in accordance with the latest scientific indications and current legislation [3]. In this context, the occupational physician works with the employer to identify any work processes that may constitute a risk. In addition, he/she identifies susceptible workers, provides information on health monitoring and on the advantages/disadvantages of vaccination/non-vaccination, and is responsible for ensuring that vaccinations are carried out.

The actual vaccination may be performed either by the occupational physician or by a qualified member of the healthcare staff under the strict supervision and responsibility of the occupational physician. It may also be delegated to the outpatient facilities of the local health agencies. A thorough vaccination history, the acquisition of proper clinical documentation and, where necessary, specific serological tests together with the implementation of vaccination programs throughout the institution can reduce the number of workers susceptible to vaccine-preventable infectious diseases with consequent benefits in terms of biological risk management. Moreover, the occupational physician should make the employer aware of the need to draw up intervention plans and to allocate adequate resources for the implementation of recommended vaccination strategies and for the monitoring of biological risk among the workers exposed. In addition, the occupational physician has to plan and implement all the diagnostic procedures and active and passive immuno-prophylactic interventions in the event of biological accidents and possible contact with transmissible pathogens. Indeed, the proper management of post-exposure prophylaxis also plays an important role in curbing the risk of infection in the workplace [6].

With regard to mandatory vaccinations in the occupational setting, Law N° 292 of 5 March 1963 concerning “obligatory anti-tetanus vaccination”, which is still in force, mandates this vaccination for some categories of workers (e.g., shepherds, agricultural workers, laborers, and refuse-collection/disposal workers). Finally, it should be borne in mind that the activity of the occupational physician is directed to safeguarding not only the health of the individual worker but also that of third parties, as stated in the International Code of Ethics for Occupational Health Professionals (ICOH) and underlined in Art. 39, subsection 1, of D.Lgs. 81/2008 [3,7].

## 3. Vaccination as a Means of Preventing the Risk of Infection in Healthcare Workers

In the 2017–2019 PNPV, one of the priority interventions specified by the Italian Ministry of Health is the promotion of an adequate vaccination strategy for healthcare workers and students in healthcare disciplines with the aim to prevent hepatitis B, influenza, measles, mumps, rubella (MMR), varicella, and pertussis infections. In this regard, it should be borne in mind that, in most cases, active immunization plays a role not only in protecting the individual worker but also (and especially) in protecting patients to whom the worker may transmit the infection [5]. Moreover, the 2010–2015 National Plan for the Elimination of Measles and Congenital Rubella (PNEMoRC), as recalled by the 2017–2019 PNPV, considers the promotion of vaccination against measles and rubella to be a priority among healthcare workers who are still susceptible [8]. Regarding measles, in 2018 in Italy, 2526 cases were reported, 115 of which involved healthcare workers [9]. Up to 30 April 2019, a total of 864 new cases of measles were reported in subjects with a mean age of 30 years; in 87% of these cases, the subjects had not been vaccinated. About 6% of these cases involved healthcare workers, while 1.7% involved school staff [10].

In Italy, vaccination against the main preventable diseases displays distinctly inadequate coverage rates among healthcare workers. This means not only that susceptible workers are insufficiently protected but also that the prevention of epidemics in hospital facilities cannot be guaranteed, as demonstrated by the outbreaks that occurred in 2017 [11,12,13].

The importance of achieving adequate vaccination coverage among healthcare workers and students of healthcare professions, particularly those who attend high-risk departments (e.g., neonatology, oncology), and also among school staff and caregivers, was further underlined by the Ministry of Health circular of 16 August 2017. This document provided the first operative instructions for the application of Decree Law N°. 73 of 7 June 2017, subsequently amended by Law N° 119 of 31 July 2017 (urgent dispositions concerning vaccine prevention). Indeed, the aforementioned ministerial circular emphasizes the need to implement vaccination campaigns against measles, mumps, rubella, whooping cough, chickenpox, hepatitis B, and influenza and to verify, when necessary, the actual status of immunocompetence [14].

During the course of their duties, healthcare workers are likely to come into contact with infected patients and material contaminated by pathogenic biological agents. For this reason, they are obviously at higher risk of contracting and spreading vaccine-preventable infectious diseases than the general population. On the basis of these considerations, the group of occupational physicians of the health agencies of the Liguria region recommends that all healthcare workers and subjects with an equivalent risk of exposure in the professional environment should undergo the vaccinations reported in Table 2 [15].

In 2018, the Emilia-Romagna Region tackled the issue of preventing occupational biological risk in the healthcare setting by drawing up the document: “Biological risk in the healthcare setting. Guidelines for the prevention of the principal blood-borne diseases (HBV, HCV, HIV) and airborne diseases (tuberculosis, measles, mumps, rubella and chickenpox): indications for the suitability of healthcare workers”, which was approved through Deliberation N°. 351 of 12/03/2018. This document, which is valid only within the Region, recalls the recommendations regarding vaccine prevention for healthcare workers expressed by the 2017-2019 PNPV (5). In accordance with these guidelines, the suitability of workers in departments identified as being at high risk, even for third parties, can only be certified after the workers have proved non-susceptible to diseases that are transmissible via air (such as chickenpox and measles) and droplets (such as mumps and rubella). The document also provides Occupational Physician with useful indications for formulating the judgment of suitability for specific tasks in the case of healthcare workers with infections due to agents transmissible through the parenteral route (HBV, HCV and HIV) or those who are not vaccinated against HBV or who are non-responders [16].

Some of the main Italian scientific societies involved in this sector have repeatedly advocated the need to promote vaccination among healthcare workers in order to control vaccine-preventable diseases. They also emphatically support the proposal of obligatory vaccination as a prerequisite for school attendance and maintain that obligatory vaccination should be extended to all staff working in school and healthcare environments [17].

## 4. Vaccination as a Means of Protecting Workers and Preventing the Risk of Infection in Non-Healthcare Occupational Settings

Other categories of workers may be exposed to biological agents whose transmission can be effectively prevented by vaccination, as specified in the 2017–2019 PNPV. Indeed, this document identifies some categories at risk and provides vaccination recommendations for each of these (Table 3) [5]. The number of occupational categories and the workers targeted for vaccination interventions may be increased according to the epidemiological context and the outcome of risk evaluation. Consequently, the provision of efficacious vaccinations by the employer may be extended.

## 5. Brief Final Remarks

Adequate knowledge of the epidemiological context and a thorough evaluation of risk at both the level of the workplace and of the individual are indispensable to appropriate consultation by the occupational physician in order to enable the employer to provide efficacious vaccinations.

In the sphere of vaccine prophylaxis, however, particular situations may emerge, such as the presence of contraindications to vaccination or cases of vaccine inefficacy (e.g., non-responders). Moreover, workers may refuse to undergo the recommended vaccinations. Such eventualities may prove particularly critical in the formulation of the judgment of suitability, especially in the absence of specific legislative references (such as obligatory anti-tetanus vaccination) or of equally efficacious, feasible alternative measures. In the light of this, it might be appropriate to undertake discussion at the national institutional level with regard to the possibility of redefining the boundaries and the prerogatives of the judgment of suitability expressed by the occupational physician who is called upon not only to guarantee the health of the worker at risk but also to safeguard third parties.

Only through close collaboration among the services of occupational medicine, vaccination clinics, and healthcare management in both hospital and territorial settings together with the implementation of vaccination programs that are agreed upon at the institutional level will it be possible to reduce the number of workers who are susceptible to vaccine-preventable diseases. Achieving this objective would yield benefits in terms of the management of biological risk in the workplace and contribute to increasing vaccination coverage rates, which in many cases are currently unsatisfactory.

In addition, health surveillance offers the opportunity to promote the culture of the prevention of infective biological risk in the workplace and elsewhere by raising awareness of the fact that vaccinations are the chief means of individual protection and collective prevention with evident benefits in the sphere of public health as well.

Pursuing the strategy of investment in disease prevention and health promotion in the occupational setting serves to develop a productive, sustainable society. In this regard, promoting a culture of informed vaccination and achieving adequate vaccination coverage rates is in the interests not only of the populations targeted for vaccination but also of the entire community in that it would help to reduce the risk of transmitting dangerous infective agents.

## Figures and Tables

**Table 1:** Classification of biological agents into four groups on the basis of their degree of hazard (infectivity, pathogenicity, transmissibility, neutralizability) in accordance with Art. 268 of Legislative Decree (D.Lgs.) 81/2008. If a biological agent cannot be assigned unequivocally to one of the four risk-groups below, it is assigned to the higher of the two possible groups considered [3].

**Group 1:**	Biological agents that display a low probability of causing disease in humans.
**Group 2:**	Biological agents that can cause disease in humans and constitute a risk for workers; unlikely to spread in the community; efficacious prophylactic and therapeutic measures are normally available (e.g., *Bordetella pertussis*, *Clostridium tetani*, *Legionella pneumophila*, influenza, *Coronavirus*, *Vibrio cholerae*).
**Group 3:**	Biological agents that can cause severe disease in humans and constitute a serious risk for workers; the biological agent may spread in the community, but efficacious prophylactic or therapeutic measures are normally available [e.g., *Mycobacterium tuberculosis*, viral hepatitis type B (HBV), hepatitis type C (HCV), human immunodeficiency virus (HIV)].
**Group 4:**	Biological agents that can cause severe disease in humans, constitute a serious risk for workers, and may display a high risk of spreading in the community; efficacious prophylactic or therapeutic measures are not normally available (e.g., Ebola, Lassa, Marburg viruses).

**Table 2:** Vaccinations recommended for healthcare workers and equivalent subjects.

Preventable Infection/Disease	Recommendation and Schedule	
Viral hepatitis type B (HBV)	Recommended for all susceptible workers Schedules available: Pre-exposure (3 doses at times 0, 1, and 6 months) Imminent pre-exposure (4 doses at times 0, 1, 2, and 12 months)	Ra
Varicella	Recommended for all susceptible workers Recommended schedule: 2 doses at times 0 and 4–8 weeks	Ra
Measles-mumps-rubella (MMR)	Recommended for all susceptible workers Recommended schedule: 2 doses at times 0 and 4–8 weeks	Ra
Influenza	Recommended for all healthcare workers One dose to be administered annually	Ra
Diphtheria-tetanus-pertussis (Tdap)	Recommended for all healthcare workers. Booster recommended: on completion of primary cycle, every 10 years for life.	Rs
Meningococcus	Healthcare workers at high risk of complications following meningococcal diseases and in particular cases of increased occupational exposure (e.g., microbiologists and researchers who handle meningococcal isolates, personnel of emergency services and infectious diseases, also according to the epidemiology and the facility risk assestment). Available vaccines and schedules: Type C conjugate vaccine (Men C) (1-dose schedule) Type A, C, W 135, and Y quadrivalent conjugate vaccine (MCV4) (1-dose schedule) Type B meningococcal vaccine (2-dose schedule at times 0 and 4 weeks)	Rs
Viral hepatitis type A (HAV)	Healthcare workers at high risk of complications following diseases due to HAV and in particular cases of increased occupational exposure (e.g., laboratory staff and researchers involved in handling, personnel in contact with HAV-infected primates, health care workers of emergency services, pediatrics, gastroenterology, infectious diseases, staff who travel to or work in countries where HAV is highly or moderately endemic, also according to the epidemiology and facility risk assestment). Recommended schedule: 2 doses at times 0 and 6–12 months.	Rs
Bacillus Calmette–Guérin (BCG)	Decree of the President of the Republic (DPR) 465/2001 drastically limited the indications for the administration of this vaccination only tuberculin skin-test (TST) negative healthcare workers at high risk of exposure to multidrug-resistant strains of tubercular bacilli or those who work in high-risk environments and cannot undergo preventive therapy because of clinical contraindications to the use of specific drugs in the case of TST conversion.	Rs

Ra = recommended for all healthcare workers; Rs = recommended for specific groups of healthcare workers.

**Table 3:** Vaccinations indicated for subjects at risk of occupational exposure. (Adapted from PNPV 2017-2019) [5].

**Anti-hepatitis A vaccination**	subjects working in contact with primates infected by the hepatitis A virus (HAV); subjects working with HAV in laboratory facilities.
**Anti-hepatitis B vaccination**	healthcare personnel newly employed by the National Health Service (NHS) and NHS staff already involved in activities at major risk of infection, particularly those working in hemodialysis, intensive care units, oncology, general and specialist surgery, obstetrics and gynecology, infectious diseases, hematology, laboratory analysis, transfusion centers, operating theaters, dental surgeries, legal medicine and autopsy rooms, emergency departments, medical assistance in prisons; persons who work, study, or carry out voluntary activities in health care; persons traveling for work reasons to geographic areas where HBV is highly endemic; staff involved in the rescue and transport of accident victims and invalids; caregivers in rehabilitation centers for drug addicts; staff of institutions for persons with physical or mental disabilities; staff involved in handling blood derivatives; religious personnel working in the healthcare sphere; members of the State Police, Carabinieri, Finance Police, Penitentiary Police, Fire Service, Municipal Police, Forestry Service; workers involved in refuse collection, transport, and disposal; tattooists and body-piercers; workers involved in the cleansing of potentially infected materials; staff of cemetery and funeral services; workers responsible for emergency and first-aid services in their workplace.
**Anti-influenza vaccination**	doctors and other staff involved in patient care; subjects performing services of primary interest to the community and categories of workers: police officers, firefighters, other socially useful categories and workers particularly exposed on account of their activities in order to reduce the negative impact on productivity; persons who, for reasons of their work, are in contact with animals that might constitute a source of infection by nonhuman influenza viruses; those involved in animal rearing or the transport of live animals, butchers, vaccinators and veterinarians.
**Ant-tick-borne meningoencephalitis (TBE) vaccination**	workers in endemic areas and in rural and woodland areas (e.g., farmers, military personnel).
**Anti-measles, mumps and rubella vaccination (MMR)**	all susceptible women exposed to a high occupational risk, particularly those working in nurseries, infant schools, primary and first-grade secondary schools; all susceptible healthcare workers.
**Anti-pertussis vaccination Tdap**	staff working in neonatal departments; nursery staff; immunocompromised individuals; all other personnel caring for newborns.
**Anti-rabies vaccination**	all workers continually at risk of exposure to the rabies virus (e.g., laboratory staff working in contact with this virus, veterinarians, biologists, stable and kennel staff, other workers in contact with potentially infected animals).
**Anti-tuberculosis vaccination (BCG)**	healthcare workers at high risk of exposure to multidrug-resistant strains of tubercular bacilli; those who work in high-risk environments who cannot, in the case of TST conversion, undergo preventive therapy because of clinical contraindications to the use of specific drugs.
**Anti-varicella vaccination**	susceptible persons working in the healthcare setting, particularly those in contact with newborns, children, pregnant women, or immunodepressed subjects; susceptible school staff.
